# Chronological Development of Cardiovascular Disease in Times of COVID-19: A Retrospective Analysis of Hospitalized Diseases of the Circulatory System and COVID-19 Patients of a German University Hospital

**DOI:** 10.3390/jcdd9100325

**Published:** 2022-09-25

**Authors:** Sebastian Griewing, Niklas Gremke, Julian Kreutz, Bernhard Schieffer, Lars Timmermann, Birgit Markus

**Affiliations:** 1 University Hospital Marburg, Philipps-University Marburg, 35043 Marburg, Hessen, Germany; 2 Institute for Health Care Management, General Business Administration, Philipps-University Marburg, Universitätsstraße 24, 35037 Marburg, Hessen, Germany

**Keywords:** COVID-19, university hospital, lockdown, circulatory system, cardiovascular

## Abstract

This study aims at examining the chronological development of hospitalized cardiovascular and COVID-19 patients and comparing the effects on related sub-disciplines and main diagnoses for pre-pandemic (2017–2019) and pandemic (2020–2021) years in the setting of a German university maximum care provider. Data were retrospectively retrieved from the hospital performance controlling system for patient collectives with main diagnosis of diseases of the circulatory system (n_Circulatory_) and COVID-19 secondary diagnosis (n_COVID-19_). The cardiovascular patient collective (n_Circulatory_ = 25,157) depicts a steady state in terms of relative yearly development of patient numbers (+0.4%, 2019–2020, +0.1%, 2020–2021). Chronological assessment points towards monthly decline during lockdowns and phases of high regional incidence of COVID-19 (i.e., 2019–2020: March −10.2%, April −12.4%, December −14.8%). Main diagnoses of congestive heart failure (+16.1% 2019/2020; +19.2% 2019/2021) and acute myocardial infarction show an increase in case numbers over the course of the whole pandemic (+15.4% 2019/2020; +9.4% 2019/2021). The results confirm negative effects on the cardiovascular care situation during the entire pandemic in the setting of a university maximum care provider. A general increase in cardiac disorders and a worrisome turn in case development of acute myocardial infarction emphasize the feared cardiovascular burden of COVID-19.

## 1. Introduction

COVID-19 (Coronavirus-Disease-19) and its rapid international spread in the spring of 2020 led to the imposition of significant restrictions on sociocultural life worldwide. A counterplay of politically enforced measures has paved the way for varying contact restrictions, recurring lockdowns and gradual easing attempts in the Federal Republic of Germany ever since. Over the course of the last two years, about 7,109,182 national COVID-19 cases (data from 30 December 2021) were confirmed by the Robert Koch Institute, the central national institution in the field of disease surveillance and prevention in Germany [1]. Internationally, the care of acute and chronic diseases has experienced noxious effects due to the pandemic [2,3,4,5,6,7]. Regular medical service provision remained almost impossible at times, as political influence was exerted on the regional and national care structure to allow for planning security and to prevent harmful capacity overload of the German health care system. Internationally, already in the early beginnings of the COVID-19 pandemic, diseases of the circulatory system were discussed as being particularly prone to the negative impact of the care situation due to cardiovascular manifestations following virus infection [7,8,9,10,11]. Cardiac conditions including acute myocardial injury, pulmonary embolism, cardiogenic shock, arrhythmias, myocarditis and even sudden heart failure, as well as ischemic stroke have been identified as health threats due to treatment complications and argue for a high burden of cardiovascular disease among COVID-19 patients [5,6,12,13,14,15,16,17]. Beyond that, heavily discussed increased risk of post-vaccination myocarditis, pericarditis or cardiac arrhythmias and severe long-term effects of COVID-19 intensify the need for monitoring patients for cardiovascular conditions to prevent a shift in acute mortality and counteract chronification of cardiac and neurological disease [5,18,19,20,21,22]. The current literature is limited to time segments of the pandemic and parts of the cardiovascular disease spectrum, neglects a clearly arranged chronological assessment and leaves out the perspective of an in-patient maximum care provider. This gave the occasion to examine and compare the entire pandemic of 2020 and 2021 with previous years of 2017 to 2019 as a university maximum service provider of the state of Hesse in Germany regarding the development of hospitalized disease of the circulatory system and COVID-19 patients. The study aims at identifying whether cardiovascular sub-disciplines and main diagnoses differ in terms of absolute and relative change in case number and how the monthly relative case development of admitted cardiovascular patients relates to dates of national lockdown and contact measures, phases of high regional COVID-19 incidence or to the discovery to new virus variants, e.g., delta and omicron virus variants. Can the feared negative effects be traced based on the development of the regional cardiovascular care situation of a central maximum service provider? 

## 2. Methods

### 2.1. Data Generation

The data for the present analysis were retrospectively retrieved from the hospital performance controlling program QlikView^®^ of Marburg University Hospital for the time period of 1 January 2017 to 31 December 2021. The system records all medical, nursing, and equipment services coded in the hospital information system. Data were collected for all patients with a main diagnosis of a disease of the circulatory system n_Circulatory_ (ICD I-diagnosis, based on ICD-10-GM 2022, International Statistical Classification of Diseases and Related Health Problems, 10th Revision, German Modification 2022) and a secondary COVID-19 diagnosis n_COVID-19_ (ICD-code “U07.1 COVID-19, virus identified”). Furthermore, the patient collective was divided in ICD-based groups for cardiovascular sub-disciplines depicted in Table 3 and the statistical protocol was repeated. The sub-group I00–02 acute rheumatic fever was neglected for further assessment as only one case was registered within the defined observation period. Moreover, a separate analysis was performed for the five most treated cardiovascular main diagnoses depicted in Table 4. The data were fully anonymized before analysis.

### 2.2. Data Analysis

The evaluation is exclusively based on methods of descriptive statistics. The focus of the study lies on the analysis of the patient collective regarding age and gender distribution, the monthly and yearly development of the overall number of hospitalized patients, the recorded main (MD) and secondary ICD diagnoses (SD), as well as standardized parameters of length of stay (LoS), patient clinical complexity level (PCCL) and case mix index (CMI). The QlikView^®^ software, developed by the company QlikTech (Radnor, United States of America), offers the possibility of displaying the clinical treatment parameters as raw data or interactive analyses. Corresponding data were generated using QlikView^®^ and then processed based on descriptive statistical methods using Excel^®^. Thus, yearly and monthly relative changes (in %) to the anteceding years were calculated for the cardiovascular patient collective (n_Circulatory_). After decomposing the general sample of n_Circulatory_ in ICD-based sub-disciplines, depicted in Table 3, the statistical protocol was repeated. Furthermore, the ten most treated cardiovascular main diagnoses and secondary diagnoses of the observation period were retrieved from QlikView^®^ (Table 2) and yearly absolute and relative figures were calculated according to the previous statistical analysis (Table 4).

### 2.3. Chronological Visualization

**Figure 1:**
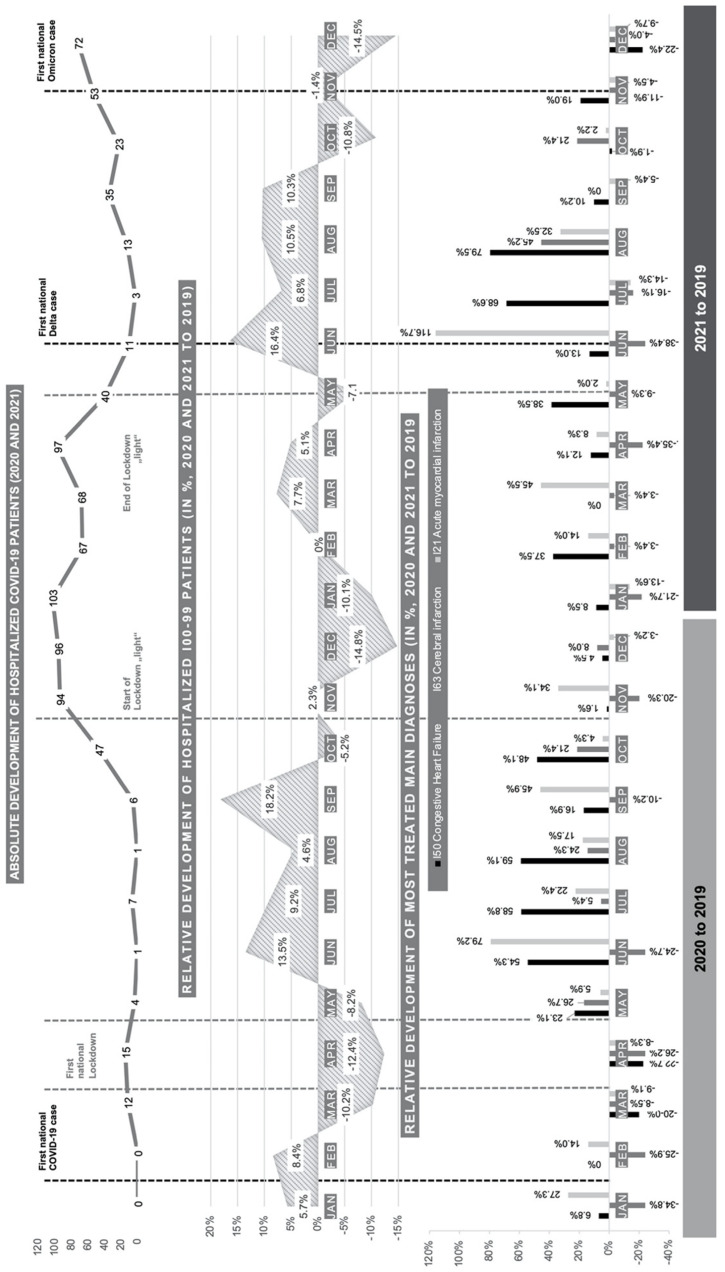
Monthly relative development of hospitalized cardiovascular and COVID-19 patients (2019–2021).

Lastly, a graphical visualization of the chronological development of hospitalized cardiovascular and COVID-19 patients was realized using Excel^®^ and Powerpoint^®^. Therefore, charts of the absolute number of in-patient COVID-19 patients per month, relative monthly change in the general cardiovascular (n_Circulatory_) and the three most treated main diagnoses were generated in Excel^®^ for the pandemic years of 2020 and 2021 (in %, in comparison to the pre-pandemic baseline of 2019). These singular charts were graphically combined in Figure 1 using Powerpoint^®^ and expanded by adding chronological dates of the beginning and end of lockdown measures, as well as the discovery of new virus variants (e.g., delta and omicron).

## 3. Results

### 3.1. Descriptive Analysis of the Patient Collectives

A total of n_Circulatory_ = 25,157 patients with cardiovascular main diagnosis were hospitalized at Marburg University Hospital within the observation period. The sample was divided into 38.72% female and 61.24% male in terms of gender distribution (0.04%, *n* = 11 cases with unspecified gender) with a mean age of 71.6 and 67.9 years. The data show a steady state in total case number development for pre-pandemic (+0.1% 2017–2018, +2,5% 2018–2019) as well as the first (+0.4%, 2019–2020) and second pandemic years (+0.1%, 2020–2021, +0.5%, 2019–2021). Regarding the in-patient-treated COVID-19 patient collective, a total of n_COVID-19_ = 868 patients were analyzed, leaving a relative increase of +107% (2020–2021) between the first and second pandemic year. The gender distribution was divided into 42.2% female and 57.8% male with a mean age of 63.2 and 62, respectively. Data of the monthly relative and absolute development of cardiovascular cases, as well as an overview of the ten most treated MD and SD within the defined observation period, are illustrated in Table 1 and Table 2.

### 3.2. Yearly Absolute and Relative Development of Cardiovascular Sub-Disciplines (2017–2021)

The yearly change in patient number for the cardiovascular sub-disciplines in relative and absolute figures and corresponding parameters for pre-pandemic (2017–2019) as well as pandemic years (2020–2021) are depicted in Table 3. Furthermore, the overall relative share of the n_Circulatory_ = 25,157 cases for the whole observation period was identified for each sub-group.

### 3.3. Monthly Relative Case Development of Cardiovascular and COVID-19 Patient Collectives (2019–2021)

The monthly case count of all in-patient cases with a main diagnosis of the circulatory system, which were admitted at the Marburg University Hospital in 2019, was used as a pre-pandemic baseline to visualize the relative monthly change in comparison to the pandemic years of 2020 and 2021. Accordingly, a visualization of the three most treated cardiovascular diseases was added to Figure 1. Table 4 provides an overview of the corresponding data. The illustration is combined with dates of lockdown and contact restriction measures chosen by the Government of the Federal Republic of Germany and dates of the first national cases of COVID-19 delta and omicron virus variants.

## 4. Discussion

### 4.1. Main Findings

The analysis illustrates a worrisome chronological development of in-patiently treated cardiovascular disease for the catchment area of Marburg University Hospital. Although the data appear to present a steady state in terms of yearly development of overall cardiovascular cases (+0.4% 2019–2021, +0.5% 2019–2021), which is within the range of year-to-year variations, the chronological assessment points towards a pattern of monthly decline in hospitalized patient number during lockdowns and phases of high regional incidence of COVID-19 (i.e., 2019–2020: March −10.2%, April −12.4%, December −14.8%). The internationally feared rise in diseases of the circulatory system due to cardiovascular complications and manifestations does not confirm on a yearly basis for the catchment area of Marburg University Hospital during the first two years of the pandemic, but monthly assessment points towards phases of catch-up effect with an increased number of cardiovascular cases following a decline (i.e., median relative increase June-September 2020 of +11.4%) [6,7,8,9,12,13,15,16]. Since the national roll-out of vaccination programs in January 2021, the cardiovascular case numbers have stabilized in terms of monthly variations (i.e., median relative increase February–May 2021 of +1.4%) followed by another phase of catch-up effect at the end of the second lockdown and decreasing regional incidence (i.e., median relative increase June–September 2021 of +10.9%). As such, the feared effect of cardiovascular post-vaccination and the long-term complications of COVID-19 does not cannot yet be visualized on a yearly basis for the development of overall regional cardiovascular disease in the care environment of Marburg University Hospital [5,18,19,20]. Nevertheless, the analysis of the sub-disciplines and most treated main diagnoses points towards varying effects of cardiovascular specialties and diseases and calls for a more nuanced illustration of the impact of the pandemic on the regional care situation.

### 4.2. Interpretation of Findings

#### 4.2.1. Comparison of Cardiovascular Sub-Disciplines and Main Diagnoses

Reported standardized parameters of LoS, CMI, PCCL and SD of the different cardiovascular sub-disciplines, which are often used to assess and compare disease severity, remain mainly unchanged in pre-pandemic and pandemic comparison. Nonetheless, reported general trends of shortening of LoS due to promotion of out-patient treatment and increased patient turnover as well as reduction in billable and CMI-relevant diagnoses and codifications can be traced back to a gradual decline in LoS, CMI and PCCL for the whole cardiovascular spectrum [23]. Moreover, data confirm a swift increase in SD in 2021 that runs through all sub-disciplines. For Marburg University Hospital, this development can be traced back to increased coding effort; nevertheless, it does not reflect on CMI.

The sub-groups of I30–52, other forms of cardiac disease (35.4%), I20–25 ischemic heart disease (22.4%), I60–69 cerebrovascular diseases (20.6%) and I70–79 diseases of arteries, arterioles and capillaries (12.1%) account for about 91% of cardiovascular patient collective, leaving congestive heart failure (I50), cerebral infarction (I63), acute myocardial infarction (I25) and atrial fibrillation and flutter (I48) as the most treated diseases, accounting for half of all in-patient cases. These disorders have also been identified to represent acute, post-vaccination and long-term COVID-19 complications [5,6,7,8,12,13,14,16,19,20,21]. Unfortunately, sub-groups with the highest share of overall case count tend to realize a relative increase in patient number during the first year of the pandemic. After two years of gradual decrease in ischemic heart disease I20–25, the case development takes a break from this favorable trend with a slight relative increase (+0.4% 2019/2020), before returning to pre-pandemic decrease levels in the second year of the pandemic (−4.7% 2019/2021). Other forms of cardiac disease I30–52 follow a trend of relative increase for the whole pandemic (+4.3 2019/2020, +6.6% 2019/2021), although the pre-pandemic year of 2019 was already characterized by a heavy increase in case numbers (+12.3% 2018/2019). By contrast, cerebrovascular diseases I60–69 depict an opposite development by realizing a decline in case numbers for the first pandemic year, followed by an increase in the second (−4.8% 2019/2020, +3.0% 2019/2021). The assessment of singular cardiovascular diseases illustrates that congestive heart failure (CHF) upholds to a trend of increase (+16.2 2019/2020, +19.2% 2019/2021), while cerebral infarction (CI) follows a trend of decrease (−9.6% 2019/2020, −9.6% 2019/2021) during the pandemic. In this context, it needs to be emphasized that acute myocardial infarction (AMI) depicts a heavy and alarming rise in case numbers for the whole pandemic after two years of consecutive decline (+15.4% 2019/2020; +9.4% 2019/2021), matching the suspected and feared consequences of the COVID-19 pandemic [12,13].

#### 4.2.2. Comparison of Chronological Development of Cardiovascular and COVID-19 Patient Collectives

On the 27 January 2020, the first national COVID-19 case was registered in Germany. Due to the swift case development, the government of the Federal Republic of Germany initiated a “hard” lockdown with complete contact restrictions on 22 March. Decisions on first relaxations of contact measures were taken on 3 May 2020. During summer, only minor restrictions were maintained, and the government reacted to a second accelerated COVID-19 case development by imposing a second “light” lockdown in early November, including softer contact measures than in the first “hard” lockdown. With the first national lockdown, Figure 1 visualizes a fierce decline in monthly relative case development of hospitalized cardiovascular patients for Marburg University Hospital. The intensity of this decline turns out to be the highest for the most treated main diagnosis of CHF (i.e., March–April 2020: −21.4%). This could be traced back to the elective care potential of the disorder and associated postponed treatment; nonetheless, acute diseases of CI or AMI also maintain an adverse and inexplicable decline. Vice versa, CHF follows a pattern of increase and catch-up effect from May 2020 onwards (i.e., median increase of +43.3% May–October 2020) that is slowed by another down shift during the second lockdown “light” (i.e., median increase of +4.8%, November 2020–January 2021) followed by another phase of acceleration (i.e., median increase of +32.4%, February–September 2021). AMI depicts a similar chronological case development over the course of the pandemic, while CI presents monthly fluctuations without a visible pattern. Figure 1 supports the second wave of COVID-19 cases to be far more intense in terms of in-patiently treated COVID-19 for the catchment area of Marburg University Hospital. While most of the literature only focuses on the first national “hard” lockdown, the presented study does not only confirm the development during the first national “hard” lockdown in the setting of an in-patient maximum service provider, but, beyond that, enables a comparison with the second “soft” lockdown. The findings confirm a decline in admissions of AMI, CHF and CI during the first phase of high incidence of COVID-19 and “hard” lockdown measures and expand these towards full chronological assessment [24,25,26,27,28,29]. The literature suggests that the initial decline in admissions was associated with the fear of getting infected during hospitalization and being confronted with overloaded care structures [24,25,27]. Although the second lockdown lasted more than three times as long as the first one and the number of in-patient COVID-19 patients of Marburg University Hospital clearly surpassed the levels of the first “hard” lockdown, the reactive decline in in-patient cardiovascular cases remained visibly lower. Beyond that, the care situation reverted to the pre-pandemic level with the initiation of the national vaccination program in early 2021. Therefore, it could be argued that fear of hospitalization during the first lockdown and other externalities, which led to the reported decline in acute cardiovascular admissions, diminished over the course of the pandemic [24,25,27]. The chronological assessment does not provide reference to the discovery of new COVID-19 virus variants having a visible effect on the care situation. However, the rise of delta as the dominant virus variant in the last quarter of 2021 is associated with another detectable downswing in cardiovascular case numbers that calls for further epidemiological analysis.

### 4.3. Limitations and Future Research

We acknowledge that the study provides a considerable number of limitations. The analysis is designed as a single center data evaluation of a regional maximum service supplier of the state of Hesse in Germany, the Marburg University Hospital. Therefore, the transferability or generalization of the proposed findings is limited, as the data are valid for rather rural care. Its retrospective design restricts the analysis to the evaluation of previously registered information of patient stays that has been recorded in the controlling program QlikView^®^. This specific way of data extraction limits the study layout to the evaluation of in-patient cases. As the data generation is based on ICD-codification, effects of fraudulent coding, misdiagnosis or possible effects due to slight changes in ICD-classification during the observation period cannot be ruled out. For these reasons, an expansion of the analysis to urban areas, an out-patient setting and multicentric data comparison would be favorable.

Furthermore, the analysis is solely based on descriptive statistical assessment. Future research needs to build up on these results by adding further variables to a study design (i.e., mortality, cardiac arrest frequency, ventilation necessity, vaccination status, etc.) to enable comparative analysis of the identified relevant disease groups (i.e., I20–25 or I30–52) or main diagnoses (i.e., AMI or CHF) and assess statistical significance by applying Poisson regression. Moreover, trend and time series analysis need to be performed on longitudinal data, once accessible, to monitor suspected long-term trends in the face of the previously discussed cardiovascular disease burden of the COVID-19 pandemic.

## 5. Conclusions

The analysis examines and compares the pandemic with the previous pre-pandemic years regarding the chronological development of the in-patient cardiovascular care of Marburg University Hospital. Internationally, cardiovascular disease has been discussed as being particularly prone to the negative impact on its care situation due to the acute cardiac and neurological manifestations of COVID-19 on the one hand and long-term effects of infection and post-vaccination complications on the other. While the general development of cardiovascular care appears to remain largely unaffected by the pandemic at first glance, the analysis suggests that effects on hospitalized cardiovascular patients diverge between sub-disciplines. The findings suggest that imposed lockdowns or contact restrictions as well as phases of high regional COVID-19 incidence have an adverse effect on the care situation with a reactive decrease in patient numbers. Nonetheless, a general increase in cardiac disorders and a worrisome turn in case development of acute myocardial infarction with a notable increase in registered cases during the pandemic emphasizes the cardiovascular burden of COVID-19 in the setting of a regional university maximum service provider. Further research needs to confirm or invalidate these findings in other settings, while it is of utmost importance to measure the effects of lockdown and contact measures as well as the discovery of new virus variants to be able to proactively act against a negative impact on acute cardiovascular care and prohibit chronification of cardiac and neurological disease.

## Figures and Tables

**Table 1:** Absolute and relative development of monthly hospitalized cardiovascular and COVID-19 patients of Marburg University Hospital, 2017–2021.

	Monthly Hospitalized Cardiovascular Patients							Monthly Hospitalized COVID-19 Patients	
Months	Absolute Figures					Relative Figures, in %		Absolute Figures	
	2017	2018	2019	2020	2021	19/20	19/21	2020	2021
Jan.	456	465	455	481	409	5.7%	−10.1%	0	103
Feb.	398	417	405	439	405	8.4%	0.0%	0	67
Mar.	451	432	441	396	475	−10.2%	7.7%	12	68
Apr.	415	423	412	361	433	−12.4%	5.1%	15	97
May	421	455	466	428	433	−8.2%	−7.1%	4	40
Jun.	370	400	377	428	439	13.5%	16.4%	1	11
Jul.	412	379	414	452	442	9.2%	6.8%	7	3
Aug.	409	415	391	409	432	4.6%	10.2%	1	13
Sep.	396	392	368	435	406	18.2%	10.3%	6	35
Oct.	376	407	461	437	411	−5.2%	−10.8%	47	23
Nov.	412	399	443	453	437	2.3%	−1.4%	94	53
Dec.	429	364	440	375	376	−14.8%	−14.5%	96	72
**Overall**	**4.945**	**4.948**	**5.073**	**5.094**	**5.098**			**283**	**585**
**in % to previous year**		**+0.1%**	**+2.5%**	**+0.4%**	**+0.1%**				**+107.0%**

**Red color indicating a decrease, green color indicating an increase.**

**Table 2:** Most common MD and SD of hospitalized patients with cardiovascular disease, 2017–2021.

**Diseases of the Circulatory System (ICD I00–99), 2017–2021**				
**Rank**	**Main Diagnoses (MD)**	**Case Number**	**Relative Share,** **in %**	**Cumulative Share, in %**
	**Overall**	**25,157**		**73.80%**
1	I50 Congestive heart failure	3365	13.38%	13.38%
2	I63 Cerebral infarction	3224	12.82%	26.19%
3	I21 Acute myocardial infarction	2896	11.51%	37.70%
4	I25 Chronic ischemic heart disease	1772	7.04%	44.75%
5	I48 Atrial fibrillation and flutter	1711	6.80%	51.55%
6	I70 Atherosclerosis	1683	6.69%	58.24%
7	I20 Angina pectoris	1414	5.62%	63.86%
8	I10 Essential (primary) hypertension	994	3.95%	67.81%
9	I35 Nonrheumatic aortic valve disease	921	3.66%	71.47%
10	I61 Intracerebral hemorrhage	587	2.33%	73.80%
	**Secondary Diagnoses (SD)**	**Overall Number**	**Relative Share of Cases, in %**	**Relative Share of Overall SD,** **in %**
	**Overall**			**36.77%**
1	I10 Essential (primary) hypertension	14,598	58.03%	6.91%
2	I25 Chronic ischemic heart disease	10,235	40.68%	5.71%
3	E78 Disorders of lipoprotein metabolism and other lipidemias	7964	31.66%	3.76%
4	Z95 Presence of cardiac or vascular implants or grafts	5548	22.05%	3.19%
5	E87 Other disturbances of the water and electrolyte balance as well as the acid-base balance	5701	22.66%	3.14%
6	I48 Atrial fibrillation and flutter	6326	25.15%	3.07%
7	Z11 Special procedures for testing for infectious and parasitic diseases	6425	25.54%	3.01%
8	E11 Diabetes mellitus, type 2	6240	24.80%	2.96%
9	N18 Chronic kidney disease	5574	22.16%	2.64%
10	I50 Congestive heart failure	4629	18.40%	2.39%

**Table 3:** Yearly absolute and relative development of cardiovascular sub-disciplines, 2017–2021.

I00–99 Diseases of the Circulatory System (According ICD-10-GM 2022)	Absolute Figures					Relative Development in %			
	2017	2018	2019	2020	2021	17/18	18/19	19/20	20/21
I05–09 Chronic Rheumatic Heart Disease	4	6	1	9	10	50.0%	−83.3%	800.0%	11.1%
LoS	10.8	16.3	33.0	18.5	14.2	Details 2021	Share of overall case count		0.2%
CMI	13.3	32.7	4.7	55.1	62,1		Median Age		
PCCL	3.5	3.7	5.0	2.9	2.3		Male (30%)		74.7
SD	13.5	14.7	27.0	18.3	16.5		Female (70%)		72.8
I10–15 Hypertension [High Pressure Disease]	267	201	251	234	197	−24.7%	24.9%	−6.8%	−15.8%
LoS	3.4	3.5	3.3	2.8	3.5	Details 2021	Share of overall case count		3.9%
CMI	0.4	0.5	0.4	0.4	0.4		Median Age		
PCCL	0.5	0.4	0.5	0.5	0.4		Male (37%)		77.0
SD	3.3	3.8	4.4	4.5	6.9		Female (63%)		70.3
I20–25 Ischemic Heart Disease	1288	1268	1198	1203	1142	−1.6%	−5.5%	0.4%	−5.1%
LoS	7.1	6.6	6.8	6.7	6.4	Details 2021	Share of overall case count		22.4%
CMI	2.3	2.2	2.3	1.9	1.9		Median Age		
PCCL	1.3	1.3	1.5	1.4	1.3		Male (70%)		70.0
SD	6.2	6.5	7.1	7.7	9.6		Female (30%)		71.7
I26–28 Pulmonary Heart Disease and Diseases of the Pulmonary Circulation	91	117	80	95	100	28.6%	−31.6%	18.8%	5.3%
LoS	9.2	8.1	6.9	7.9	7.7	Details 2021	Share of overall case count		2.0%
CMI	1.9	2.1	1.2	1.1	1.3		Median Age		
PCCL	2.0	2.3	2.0	2.2	2.0		Male (51%)		61.6
SD	7.5	8.1	7.2	7.8	9.9		Female (49%)		65.6
I30–52 Other Forms of Cardiac Disease	1529	1509	1694	1766	1806	−1.3%	12.3%	4.3%	2.3%
LoS	8.5	9.0	8.5	8.6	8.0	Details 2021	Share of overall case count		35.4%
CMI	2.7	2.9	2.7	2.2	2.2		Median Age		
PCCL	1.5	1.6	1.7	1.6	1.6		Male (60%)		69.7
SD	7.1	8.0	8.7	9.3	11.6		Female (40%)		74.2
I60–69 Cerebrovascular Diseases	1013	1051	1020	971	1051	3.8%	−2.9%	−4.8%	8.2%
LoS	9.0	10.1	10.4	9.9	9.9	Details 2021	Share of overall case count		20.6%
CMI	3.0	3,3	3.5	2.6	2.6		Medium Age		
PCCL	1.6	1,4	1.4	1.5	1.5		Male (58%)		69.5
SD	7.8	9.1	9.4	11.8	13.0		Female (42%)		73.0
I70–79 Diseases of Arteries, Arterioles and Capillaries	519	557	600	621	619	7.3%	7.7%	3.5%	−0.3%
LoS	9.8	11.0	9.6	9.2	8.9	Details 2021	Share of overall case count		12.1%
CMI	2.6	2.8	2.5	1.9	2.0		Median Age		
PCCL	1.5	1.7	1.8	1.5	1.4		Male (67%)		69.1
SD	7.5	8.5	8.8	8.8	11.0		Female (32%)		70.4
I80–89 Diseases of Veins, Lymphatic Vessels and Lymph nodes, not Elsewhere Classified	199	201	183	157	140	1.0%	−9.0%	−14.2%	−10.8%
LoS	7.7	5.5	5.8	6.0	5.3	Details 2021	Share of overall case count		2.7%
CMI	1.2	0.9	1.1	0.9	0.8		Median Age		
PCCL	1.2	0.9	1.2	0.9	0.9		Male (54%)		51.0
SD	4.8	4.6	5.3	5.2	6.8		Female (46%)		56.9
I95–99 Other and Unspecified Diseases of the Circulatory System	35	38	46	38	32	8.6%	21.1%	−17.4%	−15.8%
LoS	3.9	3.5	4.3	3.0	3.9	Details 2021	Share of overall case count		0.6%
CMI	0.5	0.5	0.6	0.4	0.4		Median Age		
PCCL	0.7	0.5	0.7	0.5	0.8		Male (49%)		67.1
SD	4.7	4.8	5.7	5.4	8.8		Female (51%)		51.7
Sum	4945	4948	5073	5094	5097	0.1%	2.5%	0.4%	0.1%
LoS	7.7	6.4	9.8	8.1	7.5				
CMI	2.0	1.7	2.1	2.0	2.0				
PCCL	1.5	1.1	1.7	1.4	1.3				
SD	6.9	5.9	9.3	8.7	10.4				

LoS = Length of Stay, in days; CMI = Case Mix Index; PCCL = Patient Clinical Complexity Index; SD = Number of Secondary Diagnoses. **Red color indicating a decrease, green color indicating an increase.**

**Table 4:** Absolute and relative development of yearly hospitalized patients of the most common main cardiovascular main diagnoses, 2017–2022.

	Absolute Yearly Development of Hospitalized Patients, 2017–2021					Relative Yearly Development of Hospitalized Patients, in %			
Main Diagnosis	2017	2018	2019	2020	2021	17/18	18/19	19/20	19/21
**I50 Congestive Heart Failure**	587	525	672	780	801	−10.6%	28.0%	16.1%	19.2%
**I63 Cerebral** **Infarction**	637	689	676	611	611	8.2%	−1.9%	−9.6%	−9.6%
**I21 Acute Myocardial Infarction**	596	572	532	614	582	−4.0%	−7.0%	15.4%	9.4%
**I25 Chronic Ischemic Heart Disease**	349	353	367	371	332	1.1%	4.0%	1.1%	−9.5%
**I48 Atrial Fibrillation and Flutter**	295	372	366	367	311	26.1%	−1.6%	0.3%	−15.0%

**Red color indicating a decrease, green color indicating an increase.**

## Data Availability

The datasets generated during and analyzed during the current study are available from the corresponding author on reasonable request.
